# Fabrication of Carbon Fiber Reinforced Aromatic Polyamide Composites and Their Thermal Conductivities with a h-BN Filler

**DOI:** 10.3390/polym13010021

**Published:** 2020-12-23

**Authors:** Min Jun Lee, Pil Gyu Lee, Il-Joon Bae, Jong Sung Won, Min Hong Jeon, Seung Goo Lee

**Affiliations:** 1 Department of Advanced Organic Materials & Textile Engineering, Chungnam National University, Daejeon 34134, Korea; irai94@o.cnu.ac.kr (M.J.L.); pilgyulee@o.cnu.ac.kr (P.G.L.); hong831@o.cnu.ac.kr (M.H.J.); 2 Carbon Fiber Thermal Textile Project Team, Research Institute of Industrial Science & Technology, Pohang 37673, Korea; ijbae68@rist.re.kr; 3 Robert Frederick Smith school of Chemical and Biomolecular Engineering, Cornell University, Ithaca, NY 14853, USA; jw2636@cornell.edu

**Keywords:** aromatic polyamide, carbon fiber, thermoplastic composite, thermal conductivity, boron nitride

## Abstract

In this study, a carbon fiber-reinforced thermoplastic composite was fabricated using a new aromatic polyamide (APA) as a matrix. Non-isothermal crystallization behaviors in the cooling process of APA resin (a semi-crystalline polymer) and composite were analyzed by using a differential scanning calorimeter (DSC). To determine the optimum molding conditions, processing parameters such as the molding temperature and time were varied during compression molding of the Carbon/APA composite. The tensile and flexural properties and morphologies of the fabricated composites were analyzed. Molding at 270 °C and 50 MPa for 5 min. showed relatively good mechanical properties and morphologies; thus, this condition was selected as the optimal molding condition. In addition, to enhance the thermal conductivity of the Carbon/APA composite, a study was conducted to add hexagonal boron nitride (h-BN) as a filler. The surface of h-BN was oxidized to increase its miscibility in the resin, which resulted in better dispersity in the APA matrix. In conclusion, a Carbon/APA (h-BN) composite manufactured under optimal molding conditions with an APA resin containing surface-treated h-BN showed a thermal conductivity more than twice that of the case without h-BN.

## 1. Introduction

Carbon fiber-reinforced polymer composites have been applied in several industrial applications, especially in automotive parts, owing to their high specific strength and modulus, as well as their excellent anti-corrosive properties and chemical resistance [1,2,3]. In particular, carbon fiber-reinforced thermoplastic polymer (CFRTP) has been actively developed in recent years. It has attracted significant attention owing to its high productivity and recyclability using a melt process. Accordingly, several studies are being conducted to apply CFRTP to various fields such as automobiles, aerospace, and defense. However, disadvantages, such as poor fiber impregnation of thermoplastic resin and a lack of resin properties [4,5,6,7,8,9,10,11,12,13,14,15,16,17,18,19,20], limits its application.

Thermoplastics such as polypropylene (PP) and polyamide (PA) are mainly used as a matrix for CFRTP. These crystalline polymers form crystals through a cooling process from a molten state. Currently, crystal nucleation, crystal shape, and size appear differently, warranting the research actively being conducted [21,22]. PA is a potential candidate for a composite matrix owing to its relatively good processability, excellent physical properties, and impact resistance. However, its physical properties are easily deteriorated due to oxidation or hydrolysis when exposed to high-temperature and high-humidity environments. This is due to the fact that the moisture absorbed by PA acts as a plasticizer and can lead to hydrolysis in severe cases, which can affect the interfacial bonding between the carbon fiber and the matrix [23]. Therefore, the demand for a new thermoplastic PA-based matrix resin is increasing, and the recently developed aromatic polyamide (APA) is a major candidate. One such APA, MXD6 (poly(meta-xylylene diamide)), is produced through the polycondensation of meta-xylenediamine and adipic acid [24,25,26]. The aromatic ring present in its backbone chain provides APA with good mechanical strength and excellent thermal stability in comparison to general aliphatic PAs such as PA 6 or PA 6.6. In addition, it has high miscibility in various resins, including PET, PP, and PE, prevents oxygen permeation, and absorbs less moisture because it has excellent moisture and gas barrier properties with low free volume and diffusion path due to the introduction of aromatic ring and polymer chain interaction stronger than PA [27]. Accordingly, if APA instead of PA is used as the matrix for CFRTP, the resultant composite material is expected to possess good mechanical properties and thermal stability. CFRTP can be produced by blending it with other resins, according to the intended application. Additionally, oxidation and hydrolysis can be controlled in high-temperature and humidity environments. Moreover, its molding process is relatively similar to that of PA; therefore, a significant change to the current production processes is not required.

Generally, in the molding process of CFRTP, the matrix thermoplastic resin has a high melt viscosity. Therefore, impregnation is a problem, and low interfacial interaction with carbon fibers can compromise physical properties of the composite as well. Thus, studies on optimizing thermo-forming conditions suitable for matrix resin properties are needed [4,5,6,7,8,9,10,11,12,13,14,15,16,17,18,19,20]. Accordingly, we intended to study compression molding conditions such as molding temperature, time, and pressure, which are suitable for aromatic PA matrices using film stacking process. We have also investigated the crystallization of the APA matrix, a semi-crystalline polymer, during its cooling process.

On the other hand, in case of applications such as the automobile underbody or battery cases, heat is generated resulting in a high-temperature environment; thus, CFRTP should be capable of dissipating heat [28]. In contrast, the polymer used as the matrix of CFRTP has low thermal conductivity, with heat-dissipation properties inferior to that of metal. To increase the thermal conductivity of the polymer matrix, research has focused on introducing functional materials such as carbon nanotubes (CNTs) and graphene, metals, or ceramics into the polymer matrix. Among these, hexagonal boron nitride (h-BN) is considered to be an excellent thermally conductive filler owing to its high thermal conductivity, low thermal expansion coefficient, and chemical stability in most organic solvents and polymers even at high temperatures. Paradoxically, its high chemical stability renders it with low miscibility in the matrix resin. Thus, its dispersibility must be improved through surface treatments such as surface oxidation, high-energy radiation, and coupling agent treatment. Comparatively, surface modification by high-temperature oxidation is simple and does not require toxic substances. It is also irreversible and stable because a functional group is introduced through covalent bonds [29]. In this study, CFRTP using APA resin was prepared by mixing surface-modified h-BN, as a thermally conductive filler to enhance the thermal conductivity of the composite.

## 2. Experimentals

### 2.1. Materials

**Figure 1:**
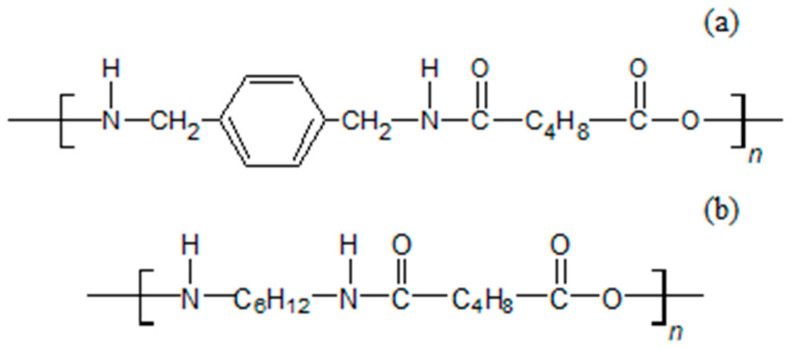
Comparison of molecular structures (**a**) aromatic polyamide (APA)(MXD6) and (**b**) Polyamide 6,6.

The APA matrix of the composite used in this study was MXD6, with the commercial name, Reny (Mitsubishi Engineering-plastics Co, Tokyo, Japan). The differences in the structure and physical properties of MXD6 and general aliphatic PA are presented in Figure 1 and Table 1. Carbon fiber, polyacrylonitrile (PAN) based Pyrofil TRW40 50L (50K) (Mitsubishi Chemical, Tokyo, Japan) was used.

### 2.2. Composite Fabrication

Carbon/APA unidirectional prepreg was prepared by the film stacking method by pressing for 1 min at 50 MPa and 270 °C through a hot compression machine (3925, Carver, Wabash, IN, USA). In addition, compression molding was performed by stacking the prepreg cross-ply with [0°/90°] to ensure optimum manufacturing conditions. The molding pressure was kept constant at 50 MPa and the molding temperature was varied (260 °C, 270 °C and 280 °C). The molding times of the Carbon/APA composites were also varied (1 min, 3 min, 5 min, and 10 min). The dimension of the prepared Carbon/APA composite was 200 × 200 × 3.2 mm and the sample codes in accordance with the molding conditions are shown in Table 2.

### 2.3. h-BN Surface Modification and Composite Fabrication

**Figure 2:**
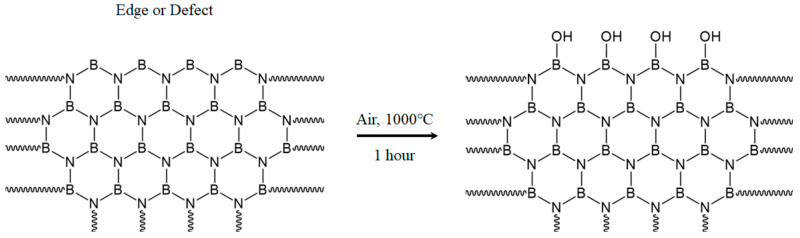
Schematic diagram of the surface treatment procedure of h-BN.

**Figure 3:**
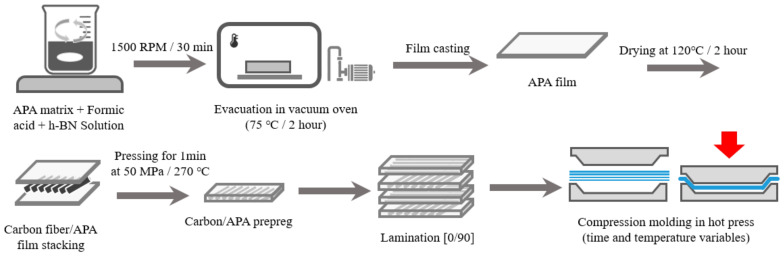
Schematic diagram of the Carbon/APA(h-BN) composite fabrication process.

To improve miscibility with APA resin, surface modification of h-BN (Figure 2) through heat treatment was performed. h-BN was heat-treated for 1 h in a furnace at 1000 °C in an air atmosphere to introduce -OH groups to the surface [29]. To incorporate surface-modified h-BN into the APA resin and Carbon/APA composite, APA films were prepared by mixing different weight ratios (3, 6, 10 wt%) of the h-BN using formic acid. The solvent was then removed in a vacuum oven for 2 h. The prepared film and carbon fiber were completely dried at 120 °C for 2 h to remove moisture and then also laminated by using the film stacking method (resin content of 40 wt%) to prepare a Carbon/APA (h-BN) composite. The schematic diagram of the whole experimental procedure of this study is shown in Figure 3.

### 2.4. Characterization

The non-isothermal crystallization behaviors of the matrix resin APA and Carbon/APA composite were analyzed while cooling at the rates of 5, 10, 20, and 40 °C/min after heating up to 300 °C by using a differential scanning calorimeter (DSC1, Mettler-Tolledo, Columbus, OH, USA). To optimize molding conditions for the Carbon/APA, the effects of the molding time and temperature on the mechanical properties and morphology were evaluated. Mechanical properties, tensile and flexural strength were evaluated according to ASTM D3039 and ASTM D790, respectively, using an UTM (AG-250Knx, Shimadzu, Kyoto, Japan), and the morphology was observed with SEM (Merlin Compact, Carl Zeiss, Oberkochen, Germany). The surface modification of h-BN was confirmed through FTIR (ATR) (Alpha-P, Bruker, Billerica, MA, USA) in the range of 4000–400 cm^−1^. The dispersion of h-BN in the APA matrix was investigated by using SEM analysis and EDS mapping (Merlin Compact, Carl Zeiss, Oberkochen, Germany) for the APA and h-BN mixture resin.

**Figure 4:**
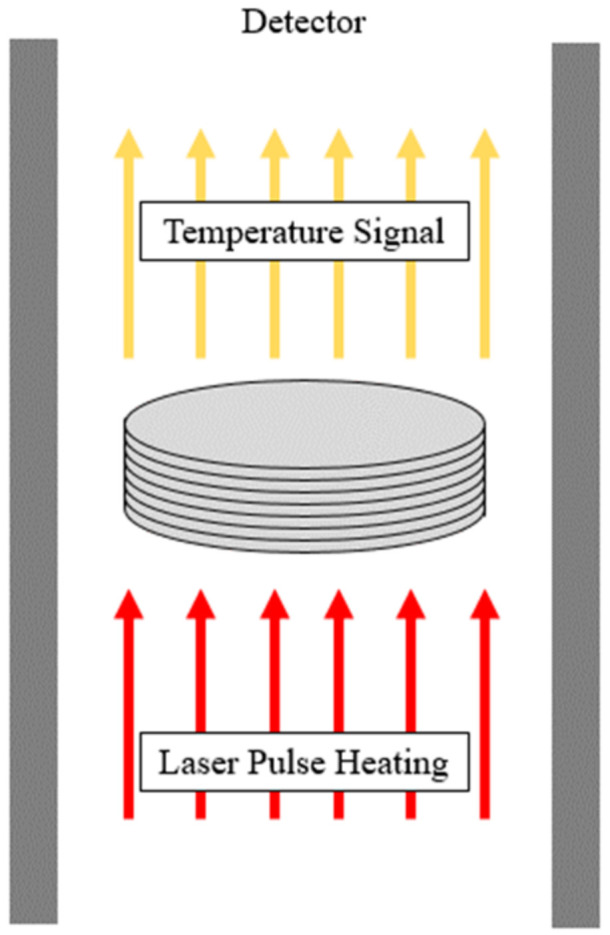
Schematic diagram of cross-plane thermal diffusivity measurement for laser flash apparatus.

The thermal conductivity of Carbon/APA (h-BN) composites was calculated using LFA (LFA-467, NETZSCH, Selb, Germany) as shown in Figure 4 and calculated using the following Equation (1):

(1) $$k=\alpha \times \rho \times C_{p}$$

where κ, α, ρ, and Cp are the thermal conductivity (W/m·k), thermal diffusivity (m^2^/s), density of specimen (kg/m^3^), and specific heat capacity (J/kg·K), respectively. The density of specimen was measured using the water displacement method. The LFA specimen measured 12.7 mm in diameter and 2 mm in thickness.

## 3. Results and Discussions

### 3.1. Crystallization Behaviors

**Figure 5:**
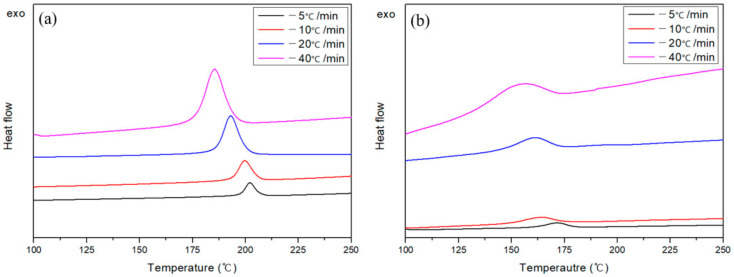
Differential scanning calorimeter (DSC) melt-crystallization curves of (**a**) modified PA and (**b**) Carbon/APA composite at different cooling rates.

Figure 5 shows a DSC curve obtained by measuring the crystallization peaks at various cooling rates to confirm the crystallization behavior of the APA matrix and the Carbon/APA composite. In both cases, the crystallization peak shifts to a lower temperature as the cooling rate increases. This is a general phenomenon in polymer crystallization. The slower the cooling rate, the longer it takes for the crystal nuclei to form; thus, the nucleation initiation occurs at a higher temperature. On the contrary, when the cooling rate is faster than the rate at which the crystal nuclei are formed, the polymer chains form crystals. Since the growth environment is insufficient, the crystallization peak appears in the low-temperature region [30]. The size of the crystallization peak seems to increase as the cooling rate increases, but the crystallization enthalpy of APA derived by integrating the calorific value over time gradually decreases: 56.26 J/g (−5 min/°C), 49.36 J/g (−10 min/°C), 48.46 J/g (−20 min/°C), and 45.61 J/g (−40 min/°C). This is believed to be due to insufficient growth of crystals at higher cooling rates. In addition, the crystallization peak of the composite appears at a lower temperature than APA. This is a result of the differences in resin flow and crystal formation due to the influence of carbon fibers [31].

**Figure 6:**
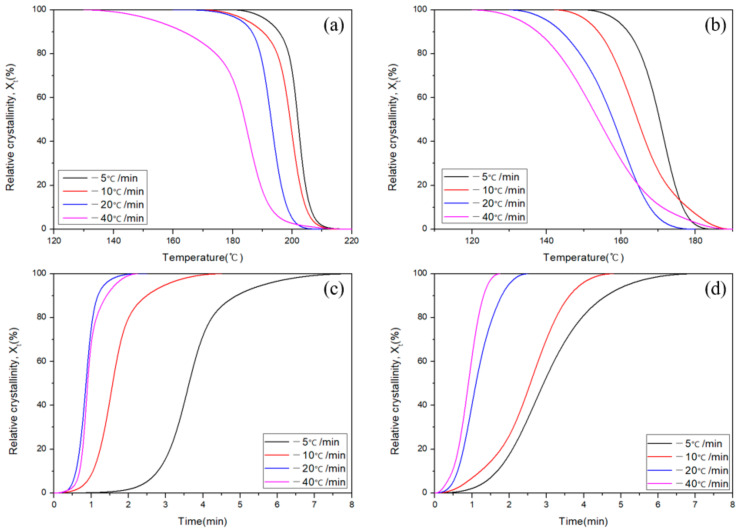
Relative crystallinity as a function of different cooling rates; (**a**) APA crystallinity over temperature, (**b**) Carbon/APA composite crystallinity over temperature, (**c**) APA crystallinity over time, and (**d**) Carbon/APA composite crystallinity over time.

**Figure 7:**
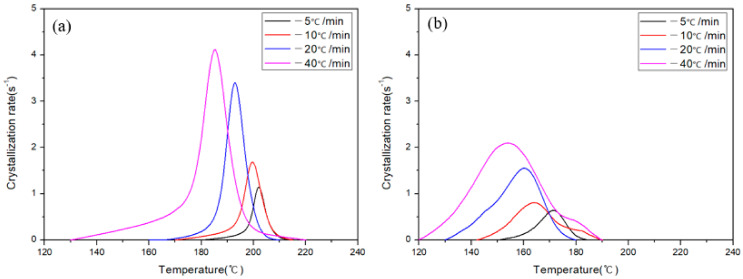
Crystallization rate of (**a**) APA and (**b**) Carbon/APA composite at different cooling rates.

Figure 6 shows the relative crystallinity of APA and Carbon/APA composite according to temperature and time. Relative crystallinity is a value expressed by integrating the heat of fusion peak according to each cooling rate and can be calculated by using Equation (2):

(2) $$X_{t}=\frac{\int_{T_{0}}^{T_{t}}\left(\frac{{dH}_{c}}{dT}\right)dT}{\int_{T_{0}}^{T_{\infty }}\left(\frac{{dH}_{c}}{dT}\right)dT}$$

where *T*_0_ is the temperature at which the crystals started to form, *T*_∞_ is the temperature at which the crystallization was complete, *T_t_* is the temperature at time *t*, and *H_c_* represents the heat of crystallization. The progress of crystallization at different cooling rates is shown in Figure 6a,b. The relative crystallinity derived by Equation (2) and shown in Figure 6a,b can be expressed as the relative crystallinity over time by plotting it against the time parameter (Figure 6c,d). As the cooling rates of both APA and Carbon/APA composites increase, the crystal nucleation temperature and the time taken to complete crystallization tend to decrease. The crystallization rate can be expressed by differentiating the relative crystallinity over time (Figure 6c,d), and is shown in Figure 7. As the cooling rate increases, the peak of the crystallization rate increases. Accordingly, the time at which crystallization completes is shortened.

The overall kinetics for crystallization process in polymers can be well described by using the Avrami Equation (3). In this study, to investigate the effects of carbon fiber on the crystallization behavior of APA, the Avrami parameter was calculated for the APA resin and Carbon/APA composite:

(3) $$1-X_{t}=\exp\;(-Kt^{n})$$

where *n* is the Avrami exponent, *K* is the overall crystallization rate constant, *X_t_* is the relative crystallinity, and *t* is the crystallization time [32]. As results derived from Equation (3), average values of n of APA resin and Carbon/APA composite were calculated as 3.145 and 2.601, respectively. Therefore, it can be considered that crystal grows three dimensionally in the APA resin, though has a two-dimensional shape in the Carbon/APA composite. This result might be caused by the difficulties in crystal growth of composite for three-dimensional structure due to the compactly arranged carbon fibers. Moreover, it seemed that a carbon fiber did not act as a nucleating agent in the APA matrix.

### 3.2. Mechanical Properties of Composites with Moding Conditions

**Figure 8:**
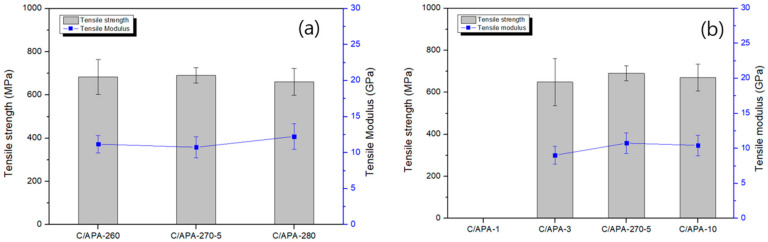
Tensile strength and modulus of Carbon/APA manufactured under different molding conditions: (**a**) molding time and (**b**) molding temperature.

**Figure 9:**
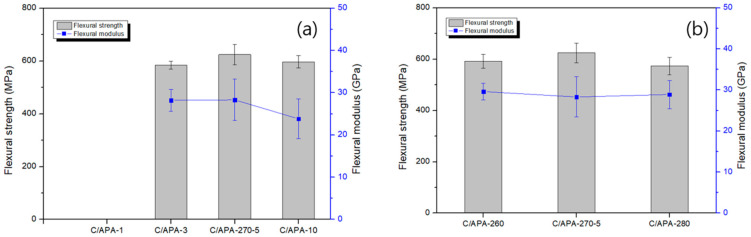
Flexural strength and modulus of Carbon/APA manufactured under different molding conditions: (**a**) molding time and (**b**) molding temperature.

Figure 8 and Figure 9 show the results of tensile and flexural strength of the carbon/APA composite manufactured under different molding conditions. It is to be noted that during specimen processing of the composite with the molding time of 1 min, interlayer separation occurred; thus, a measurement was not possible. This can be attributed to the lack of time for interfacial bonding due to melting of the matrix between the lamina. The highest values of tensile and flexural strength were obtained at a molding temperature and time of 270 °C and 5 min, respectively. It is considered that both fiber impregnation and resin properties influence the mechanical properties. In particular, the flexural properties of the Carbon/APA composite in this study were approximately twice as high as those of the Nylon 6 composite with the same carbon fiber [33].

### 3.3. Morphologies of Carbon/APA Composites

**Figure 10:**
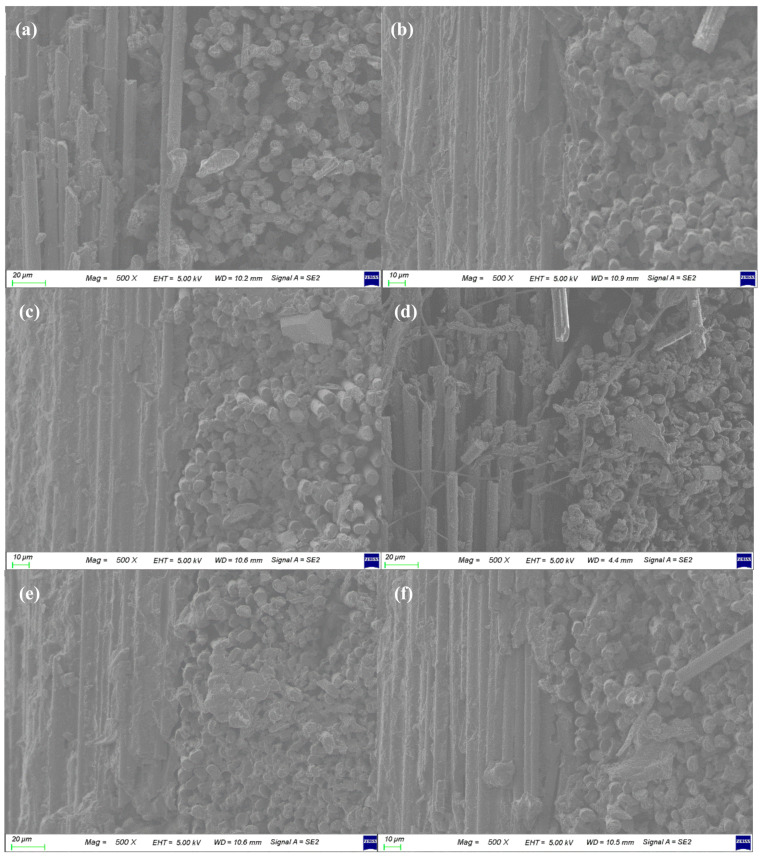
SEM images of cross-sectional surfaces of the Carbon/APA at different molding conditions; (**a**) C/APA-260, (**b**) C/APA-270-5, (**c**) C/APA-280, (**d**) C/APA-1, (**e**) C/APA-3, (**f**) C/APA-10.

The SEM images of the interface between the [0°/90°] cross-ply of the Carbon/APA are shown in Figure 10. When comparing the cross-sectional surface images of C/APA-260 (Figure 10a), C/APA-270-5 (Figure 10b), and C/APA-280 (Figure 10c) manufactured at different temperatures, the adhesion between the interfaces of C/APA-260 was poor, but improved at a higher temperature. However, in case of C/APA-280, the filament invaded the boundary between the ply, and warped. At 260 °C, the flowability between the matrices is insufficient, while at 280 °C, the flow is active and is considered to have some influence on the array of filaments. When comparing the cross-sectional surface images of C/APA-1 (Figure 10d), C/APA-3 (Figure 10e), C/APA-270-5 (Figure 10b), and C/APA-10 (Figure 10f) manufactured at different times, C/APA-1 and C/APA-3 exhibited defects or void and interface that was either poor or inadequately formed. In the case of C/APA-270-5 and C/APA-280 specimens, relatively good interface was made, but in the case of C/APA-10, the filament invaded the boundary between the ply. These results indicate that molding times of 1 min and 3 min are too short for melt bonding to occur, and that are too long to be exposed to the flow of the matrix when molding for 10 min. From the results of cross-sectional morphology observation and mechanical properties, a processing condition of C/APA-270-5 was considered to be relatively proper for the Carbon/APA composite molding in this study.

### 3.4. Theaml Conductivities of Carbon/APA(h-BN)

**Figure 11:**
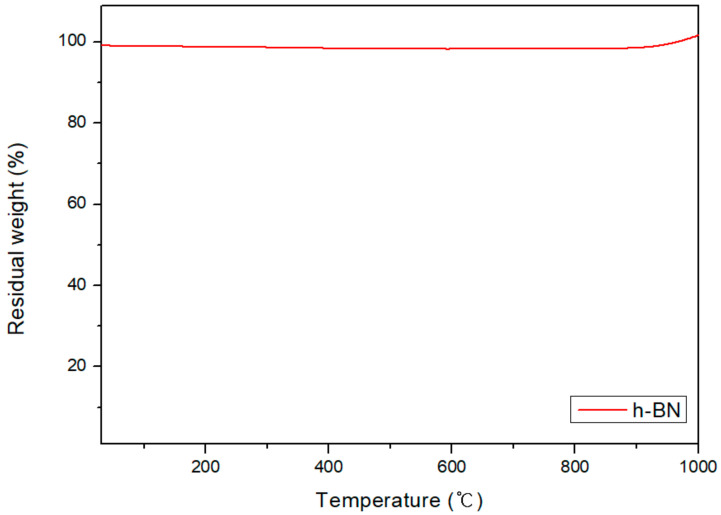
TGA thermograms of pure h-BN.

Figure 11 shows the TGA result of h-BN under heat treatment. The weight of h-BN appears to slightly decrease with increasing temperature. The trend began to reverse at approximately 900 °C, and increased by 3% at 1000 °C compared to the initial weight. It is considered that the weight increases as h-BN is oxidized from approximately 900 °C onwards (Figure 2). Therefore, heat treatment of h-BN at 1000 °C can lead the oxidation of its surface.

**Figure 12:**
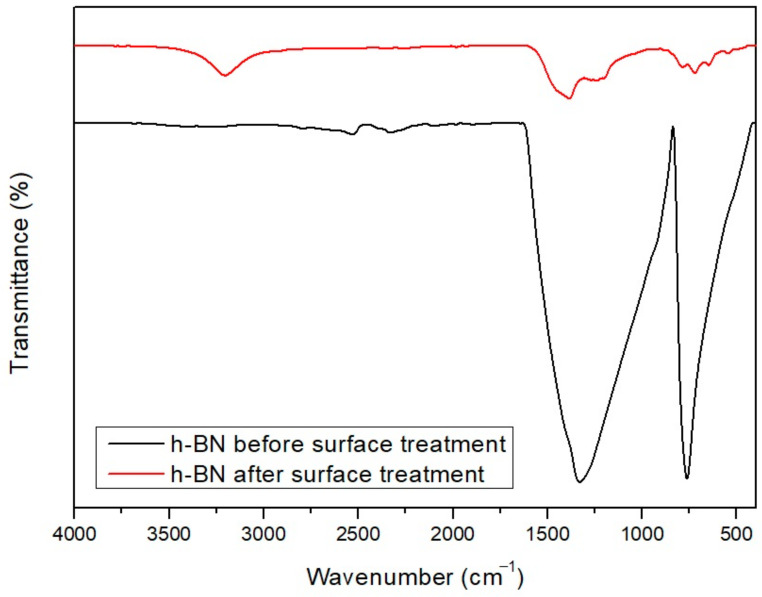
FT-IR spectrum of pure h-BN and surface-modified h-BN.

Figure 12 shows the FT-IR peak of h-BN before and after surface modification through heating at 1000 °C; common peaks appear around 780 cm^−1^ and 1350 cm^−1^ before and after heating are observed. These represent out of plain vibration of the B-N-B bond, and in plain stretching of the B–N bond, respectively. New peaks are formed at 720 cm^−1^, 1200 cm^−1^ and 3200 cm^−1^ in h-BN after oxidation. These peaks represent B–O, BN–O, and B–OH, respectively, confirming surface modification (Figure 2) [34].

**Figure 13:**
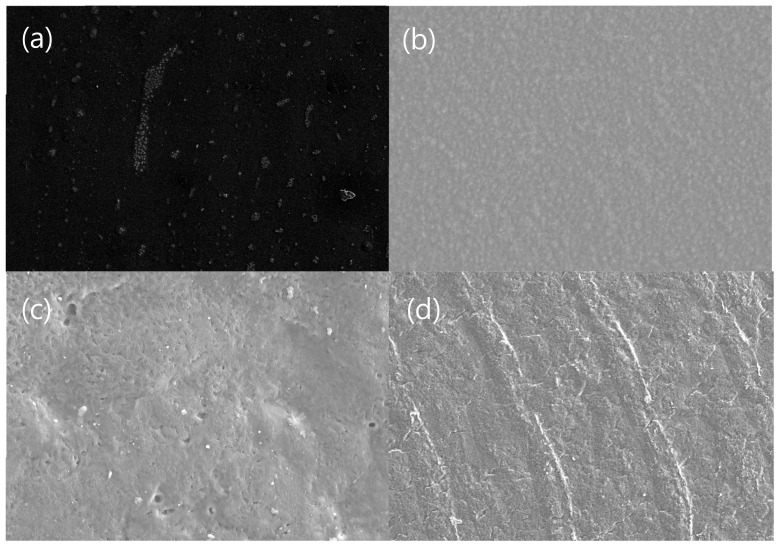
SEM images of the APA surface with different contents of h-BN; (**a**) 10 wt% of non-surface-treatment h-BN, (**b**) 3 wt%, (**c**) 6 wt%, (**d**) 10 wt%.

**Figure 14:**
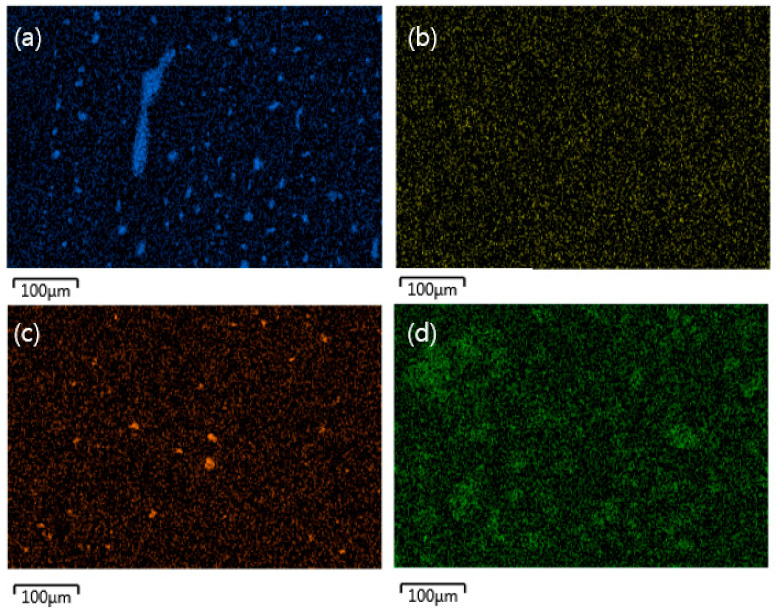
EDS mapping image of the APA surface with different contents of h-BN: (**a**) 10 wt% of untreated h-BN; (**b**) 3 wt%; (**c**) 6 wt%; and (**d**) 10 wt% of surface treated h-BN.

SEM images and EDS mapping pictures for Boron element after introducing h-BN into APA resin are shown in Figure 13 and Figure 14, respectively. In the case of APA-containing pure h-BN, significant agglomeration was observed. However, when surface-modified h-BN was added, it showed a relatively dispersed state. In addition, it was verified that when the amount of h-BN was increased, a slight aggregation of the h-BN particles was exhibited.

**Figure 15:**
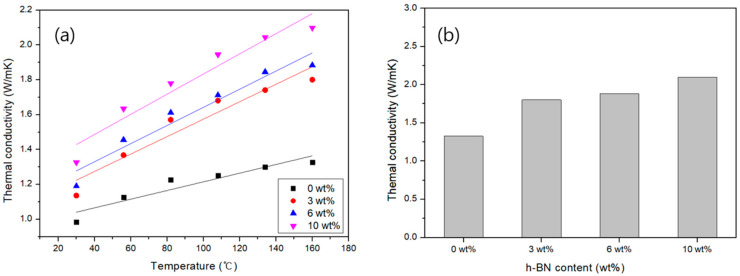
Thermal conductivities of Carbon/APA (h-BN) with different wt% of h-BN (**a**) with temperature, and (**b**) at 160 °C.

The results of measuring the thermal conductivity of Carbon/APA(h-BN) at different temperatures are shown in Figure 15. As the temperature increases, the thermal conductivity of the composite tends to increase (Figure 15a). It is apparent that adding h-BN improves the thermal conductivity. At 10 wt% h-BN, the thermal conductivity is more than twice when compared to 0 wt%, which can be attributed to the inclusion of high thermally conductive h-BN. Figure 15b specifically shows the effect of increasing h-BN content on thermal conductivity at 160 °C. It is confirmed that the thermal conductivity increases as the h-BN content increases. Therefore, it is evident from these results that Carbon/APA (h-BN) composites with improved thermal conductivity were successfully produced. It can be concluded that these composites can be used as a heat dissipating material in automobile structures.

To examine the effect of h-BN on the Carbon/APA composite under optimal conditions of 5 min heating time, temperature of 270 °C and pressure of 50 MPa, a composite was fabricated using APA with 10 wt% h-BN. Its tensile and flexural properties are shown in Table 3. There was no significant difference in either the tensile or flexural properties, but the strength slightly decreased and the modulus tended to slightly increase. This appears to be because the matrix resin becomes more rigid due to the addition of inorganic h-BN powder. As the addition of h-BN caused no significant change in the properties of the composite, it is expected that the Carbon/APA (h-BN) composite produced in this study will be used in automotive structural parts with heat-dissipation properties.

## 4. Conclusions

In this study, the manufacturing conditions and properties of carbon fiber composites with an APA matrix, and the improvement of the thermal conductivity of the composite were studied. It was proposed that APA can serve as a better alternative to the conventional polyamide matrix. The following conclusions were drawn from this study.

In the analysis of non-isothermal crystallization of APA resin, the behavior according to the cooling rates was different from that of the conventional Nylon matrix. From the Avrami parameter analysis, it can be considered that crystal grows three dimensionally in APA resin, but its growth proceeds two-dimensionally in the Carbon/APA composite. The presence of carbon fiber did not significantly affect the crystallization of the APA resin.

The optimum molding conditions during compression molding of Carbon/APA composite were 270 °C for 5 min and a pressure of 50 MPa. Composites prepared at these conditions showed relatively good mechanical properties and morphologies compared to conventional polyamide composites. This is thought to be the result of the high mechanical properties of the APA matrix.

In order to improve the thermal conductivity of the composite, h-BN was introduced as a filler. The surface of h-BN was treated with high-temperature oxidation to increase miscibility in the APA matrix, which resulted in good dispersion. The Carbon/APA (h-BN) composite showed thermal conductivity more than twice than that of composites without h-BN. As the addition of h-BN caused no significant change in the properties of the composite, it is expected that the Carbon/APA (h-BN) composite will be used as automotive structural parts for heat dissipation.

## Figures and Tables

**Table 1:** Comparison of properties of APA(MXD6) and conventional polyamide (PA)

Properties	Units	APA(MXD6)	PA6	PA66
Water absorption	%	5.8	11.5	9.9
Deflection temperature under load	°C	96	57	60
(Melting point) Tm	°C	243	225	268
Tensile strength	MPa	99.0	61.8	76.5
Tensile modulus	GPa	4.7	2.5	3.1
Flexural strength	MPa	157	123	127
Flexural modulus	GPa	4.4	2.4	2.9

**Table 2:** Sample code of Carbon/APA composites prepared at different molding conditions.

Sample Code	Molding Temperature/Time	Sample Code	Molding Temperature/Time
C/APA-260	260/5	C/APA-1	270/1
C/APA-270-5	270/5	C/APA-3	270/3
C/APA-280	280/5	C/APA-270-5	270/5
-	-	C/APA-10	270/10

**Table 3:** Mechanical properties of Carbon/APA composites according to the inclusion of h-BN (10 wt%).

	C/APA-270-5	C/APA(h-BN)-270-5
Tensile strength (MPa)	690.6	681.2
Tensile modulus (GPa)	10.8	12.7
Flexural strength (MPa)	624.3	590.4
Flexural modulus (GPa)	28.3	33.4
